# Modeling the Amino Acid Effect on Glucagon Secretion from Pancreatic Alpha Cells

**DOI:** 10.3390/metabo12040348

**Published:** 2022-04-13

**Authors:** Jan Zmazek, Vladimir Grubelnik, Rene Markovič, Marko Marhl

**Affiliations:** 1 Faculty of Natural Sciences and Mathematics, University of Maribor, 2000 Maribor, Slovenia; jan.zmazek@um.si (J.Z.); rene.markovic@um.si (R.M.); 2 Faculty of Electrical Engineering and Computer Science, University of Maribor, 2000 Maribor, Slovenia; vlado.grubelnik@um.si; 3 Faculty of Education, University of Maribor, 2000 Maribor, Slovenia; 4 Faculty of Medicine, University of Maribor, 2000 Maribor, Slovenia

**Keywords:** diabetes, hormone secretion, glucose, amino acid, cAMP

## Abstract

Type 2 Diabetes Mellitus (T2DM) is a burdensome problem in modern society, and intensive research is focused on better understanding the underlying cellular mechanisms of hormone secretion for blood glucose regulation. T2DM is a bi-hormonal disease, and in addition to 100 years of increasing knowledge about the importance of insulin, the second hormone glucagon, secreted by pancreatic alpha cells, is becoming increasingly important. We have developed a mathematical model for glucagon secretion that incorporates all major metabolic processes of glucose, fatty acids, and glutamine as the most abundant postprandial amino acid in blood. In addition, we consider cAMP signaling in alpha cells. The model predictions quantitatively estimate the relative importance of specific metabolic and signaling pathways and particularly emphasize the important role of glutamine in promoting glucagon secretion, which is in good agreement with known experimental data.

## 1. Introduction

Diabetes is a significant public health problem worldwide, with global prevalence in 2019 estimated to be 9.3% and rising to 10.9% by 2040 [1]. Diabetes causes several microvascular and macrovascular complications, such as blindness, heart attack, stroke, kidney failure, and lower-limb amputations, and is also among the top 10 causes of death in adults. Type 2 Diabetes Mellitus (T2DM) accounts for ~90% of diabetes patients. It is generally accepted that T2DM initially results from insulin resistance, followed by the decreased ability of the pancreatic beta cells to produce sufficient insulin output. However, T2DM is increasingly recognized as a bi-hormonal disease caused by glucagon excess and insulin deficiency [2,3,4]. Therefore, it is not unexpected that the focus of research, long limited to studying the mechanisms of glucose-stimulated insulin secretion (GSIS) by beta cells, is shifting to glucagon-producing alpha cells. Moreover, while the mechanisms behind beta cell insulin secretion are largely understood by the »consensus model« [5], which is essentially based on the mechanism of beta cell energy sensing, the mechanisms that trigger alpha cell glucagon secretion are still obscure.

Pancreatic alpha cells are found both at the mantle and the core of the islets of Langerhans and make up ~35–40% of the human islet cells [4,6]. It has been shown that glucose inhibits glucagon secretion by a direct effect on alpha cells [7]. The glucose-stimulated glucagon secretion (GSGS) results from the increased glycolytic flux during hypoglycemic conditions, with its extent limited by the glucose-sensing glucokinase enzyme [8,9]. The catabolic pathways increase ATP production and intracellular concentration, coupled with membrane potential (MP) by the K_ATP_ channels conductance [10]. The closure of K_ATP_ channels results in lower MP amplitude, and since glucagon exocytosis depends primarily on Ca^2+^ entry through P/Q-type voltage-gated calcium channels (VGCC) with high activation threshold, hyperglycemia reduces Ca^2+^ entry [11]. Therefore, the overall intrinsic modulation of glucagon secretion strongly resembles the GSIS, but the effect is reciprocal. However, the model of GSGS is exclusively limited to the effects of plasma glucose elevation on alpha cells, while there is still an ongoing debate as to whether glucose is genuinely the primary modulator of glucagon secretion. Namely, it is increasingly acknowledged that alpha cells respond to various stimuli, such as free fatty acids (FFAs) and amino acids (AAs). Furthermore, paracrine signals from neighboring beta and delta cells, incretins from distant tissues, and neuronal stimulation play a significant role in glucagon secretion, probably depending on the physiological context [4].

The contribution of glucose and other nutrients to intrinsic mechanisms of glucagon secretion was estimated in our previously published computational models of alpha cell metabolism [12,13,14,15]. The results introduce some critical aspects of the intrinsic mechanisms of alpha cell activation. As suggested by Schuit et al. [16], the anaerobic nature of the alpha cell, which is characterized by high expression of lactate dehydrogenase (LDH) and consequent high lactic acid output, may play a critical role in switching glucagon secretion, which is confirmed by the model results [12]. The results also indicate that FFA oxidation enables glucagon secretion by ensuring sufficient ATP production, especially under hypoglycemic conditions when the glucose oxidation pathway is largely inhibited. Moreover, mitochondrial dysfunction in alpha cells contributes significantly to the dysregulation of glucagon secretion in T2DM, further highlighting the role of intrinsic mechanisms of glucagon exocytosis [13,14]. However, recent studies suggest that the role of intracellular Ca^2+^ in the intrinsic mechanisms of glucagon secretion may be overestimated [4,17]. Instead, experimental data suggest that Ca^2+^-independent changes in subplasmalemmal cyclic-AMP (cAMP) concentration correlate strongly with changes in glucagon release [18,19,20]. To incorporate these findings, our recently proposed computational model includes glucose-induced modulation of cAMP levels, elicited by changes in the metabolic mediators CO_2_ and lactic acid [15]. The model is based on the ability of bicarbonate (HCO_3_^−^), which is in Henderson-Hasselbalch equilibrium with CO_2_ and lactic acid, to modulate the activity of soluble adenylyl cyclases (sACs) [21,22]. The contribution of the cAMP pathway to the intrinsic modulation of glucagon secretion was estimated to be approximately 60%.

Several studies established a strong link between AA and glucagon metabolism in recent years. It was proposed that glucagon and AAs are linked through the liver-alpha-cell axis, with an elevation of AAs inducing alpha cell hyperplasia and hyperglucagonemia due to disturbances in hepatic glucagon signaling [23,24,25,26,27,28]. Which specific AAs participate in the liver-alpha-cell axis is still a matter of debate, but it has been suggested that alanine, arginine, cysteine, and proline are involved [29]. Apart from the liver-alpha-cell axis, AAs induce glucagon secretion directly, and some authors hypothesize that AAs may even be the primary nutrient for stimulating glucagon secretion [30,31]. The physiological role of AAs in stimulating glucagon secretion [32,33] is most likely to prevent hypoglycemia by increasing AA catabolism and liver gluconeogenesis after protein ingestion, counteracting the anabolic effects of insulin, since AAs also stimulate beta. AAs have various potencies to stimulate alpha cells and therefore modulate glucagon secretion via separate, mostly unknown mechanisms. Alanine has a potent effect on glucagonemia, which is not surprising since it is the primary AA derived from proteolysis in muscle [34] and can be rapidly converted to the gluconeogenic substrate pyruvate. A semi-essential AA arginine also plays a role in stimulating glucagon release [31,35,36], in addition to its physiological role in modulating immune function, contributing to wound healing, regulating vascular tone, insulin sensitivity, and endothelial function during periods of maximal growth, severe stress, and injury [37]. However, arginine plays a lesser role as a postprandial AA influencing glucagon secretion in the short term.

While alanine and arginine stimulate alpha cells, the most potent AA in postprandial stimulation of glucagon secretion is most likely glutamate [31,35,36,38,39,40,41]. Specifically, it is strongly suggested that alpha cells are the site of glutamate production in the endocrine pancreas because of their high glutaminase expression [42,43]. Alpha cells also express vesicular glutamate transporters, which are found on the glucagon-containing secretory granules of alpha cells, enabling the co-release of glucagon and glutamate [44]. The co-released glutamate has been found to be a positive autocrine signal for glucagon secretion, acting on ionotropic glutamate receptors (iGluRs) [45]. On the other hand, glutamine, the precursor of glutamate, is the AA preferred for transport in the blood [46,47] and is consequently the most abundant [48]. While the ingestion of a protein-rich meal elicits an increase of virtually all AAs, the increase in absolute glutamine concentration is the greatest [48]. It also plays a central role in nitrogen metabolism [46], and its concentration increases during prolonged fasting due to increased hepatic output. Due to glutamine’s vital physiological role in postprandial metabolism, the present study complements the existing computational model [15] with the effects of plasma glutamine levels on glucagon output from alpha cells. The effect of glutamine, reflecting the general AA status, is roughly modeled by its conversion to glutamate, co-release of glutamate with glucagon, increased interstitial glutamate concentration, and glutamate action on iGluRs. Since relative glucagon secretion (RGS) does not drop 40% below maximal, even during hyperglycemia [12,13], the triggering signal for glucagon granule exocytosis persists and ensures constant co-release of glutamate in the interstitial space, mirroring the plasma glutamine concentration.

Finally, the model incorporates the autocrine effect of increased glucagon output by including the production of intracellular cAMP due to stimulation of transmembrane adenylyl cyclases (tmACs) bound to the glucagon receptor (GcgR). Thus, with GSGS and AA action mechanisms on glucagon secretion, the model covers a large portion of physiological conditions. We compare our model predictions with published experimental data on glucagon secretion during AA-enhanced GSGS [31,35,36,45].

## 2. Results

We present the results on glucose- and AA-dependent RGS obtained with the computational model introduced in Section 3 and illustrate the fundamental mechanisms behind the glucose- and AA-induced changes in MP oscillations, intracellular cAMP concentrations, and their influence on RGS.

### 2.1. Glucose- and AA-Dependent RGS

**Figure 1:**
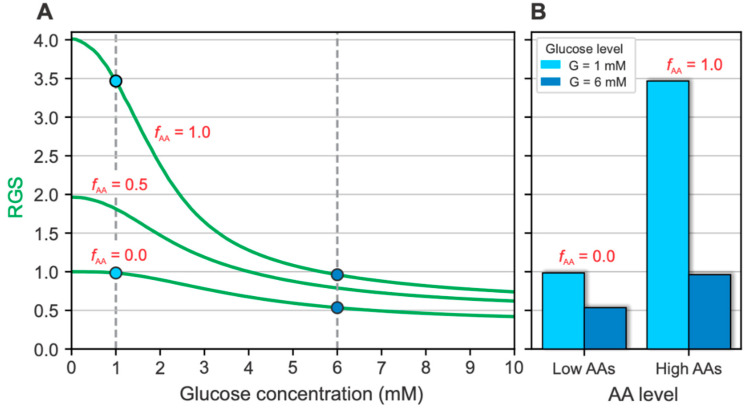
The influence of AAs on the GSGS. (**A**) Glucose-dependent RGS at various relative AA concentration levels. The results are normalized to the RGS at low AA concentration levels. The addition of high AA concentration raises the glucose-dependent RGS curve. (**B**) The comparison of the relative change of RGS at 1 mM and 6 mM glucose concentration during low- and high-AAs levels. The increase is most prominent at 1 mM glucose concentration (light blue bars, up to ~3.5-fold increase). On the other hand, the increase during 6 mM glucose concentration is less pronounced (dark blue bars, ~2-fold increase).

RGS at different glucose- and AA levels is shown in Figure 1. The computational analysis was performed for the interval of plasma glucose concentration between 0 and 10 mM and for low ($f_{AA}=0$), medium ($f_{AA}=0.5$), and high ($f_{AA}=1$) AA concentrations. The relative AA levels represent physiological levels of the AA concentration, where $f_{AA}=0$ and $f_{AA}=1$ characterize the AA concentration occurring in the fasting state and during a protein-rich meal, respectively [35,49].

Figure 1A shows the glucose-dependent RGS at different relative AA levels. The RGS curves are shown relative to the RGS value at G=0, presented by [50] and our previous works [12,13,15]. While $f_{\mathrm{AA}}$ corresponds to physiological AA levels, ranging from 0 to 1, hypoaminoacidemic levels (i.e., subphysiological AA concentration in plasma) can also be simulated by negative values of $f_{\mathrm{AA}}$. In contrast, hyperaminoacidemic levels (i.e., supraphysiological AA concentration in plasma) can be represented by $f_{AA}>1$.

In Figure 1B, the relative changes in RGS during the addition of a solution with a high AA concentration are compared. The changes are shown separately at fixed plasma glucose concentrations of 1 and 6 mM. During the addition of the high AA concentration at 1 mM glucose, RGS increased 3.5-fold. In contrast, the addition of the high AA concentration at 6 mM glucose resulted in a smaller but still convincing 2-fold increase. These results are compared with the experimental observations of Zhang et al. [35], who measured ~4-fold and ~2.2-fold increases at 1 mM and 6 mM glucose concentrations, respectively. The low-concentration (2 mM) AA mixture contained eight different AAs in the experimental study, with glutamine representing 25% of the AA content. On the other hand, the high-concentration (8 mM) AA mixture contained the same relative content (~33%) of alanine, glutamine, and arginine. Similarly, in an older study by Östenson and Grebing [39], a 2-fold increase in glucagon release was observed after adding glutamine in the presence of 5.5 mM glucose.

These results are broadly consistent with experimental data, demonstrating that AAs indeed play an essential role in triggering the glucagon response of pancreatic alpha cells. Between the extreme high-glucose-low-AA and low-glucose-high-AA states, the glucagon secretion rate can increase up to ~7-fold. Moreover, recent experimental results suggest that AAs may play a similar or even more critical role than glucose in responding to metabolic status [30,31].

### 2.2. The Intracellular Mechanisms behind Glucose- and AA-Dependent RGS

The RGS is modulated by fluctuations in intracellular Ca^2+^ and cAMP concentrations. In addition to intracellular ATP concentration and interstitial glutamate concentration, distal pathways of cAMP signaling also modulate Ca^2+^ entry. Consequently, the behavior of intracellular mechanisms behind the glucose- and AA-elicited RGS is complex. In this subsection, we present the basic intracellular mechanisms by which AAs increase the magnitude of glucose-stimulated glucagon release, including changes in MP dynamics and changes in cAMP signaling.

#### 2.2.1. Effects of MP Oscillations on RGS

**Figure 2:**
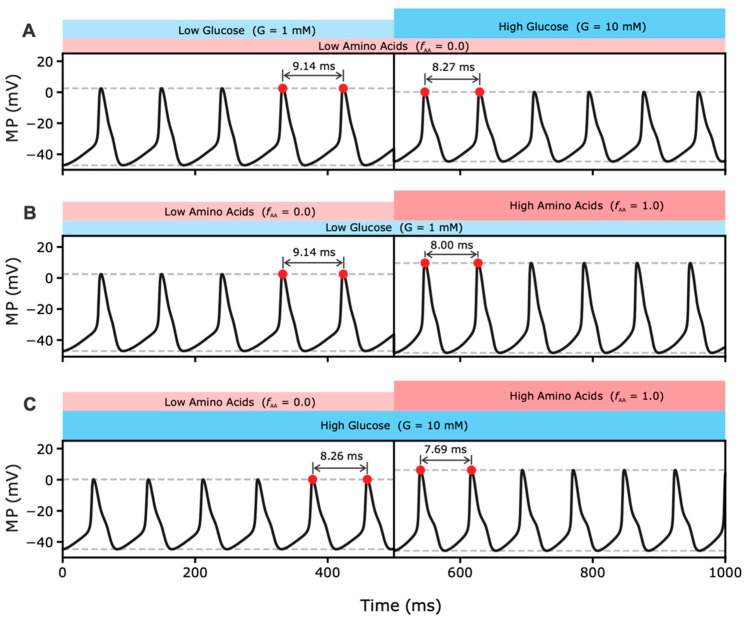
Dynamics of MP at different glucose and AA concentrations. (**A**) At high glucose concentration, the amplitude of the MP oscillations decreases and the frequency increases. (**B**) At low glucose concentration, the increase in AA concentration increases both amplitude and frequency of MP oscillations. (**C**) At high glucose concentration, the increase in AA concentration increases both the amplitude and frequency, but the change is not as significant.

The computational model presented assumes a good correlation of plasma AA concentration, especially of glutamine, with glutamate concentration in the interstitial space because alpha cells synthesize and co-secrete glutamate with glucagon. As described in Equations (6)–(8), the binding of glutamate to iGluR directly affects the dynamics of MP by modulating cation fluxes across the plasma membrane and increasing intracellular Ca^2+^ concentration and RGS. Figure 2 shows the direct effects of AAs on the dynamics of MP at different glucose concentrations in a time frame of 500 ms.

Figure 2A shows the effect of glucose on the dynamics of MP at low AA. This effect of glucose on MP oscillations is somewhat contradictory, as the higher frequency increases while the lower amplitude decreases Ca^2+^ entry. However, the effect of the lower MP amplitude predominates and results in lower RGS. Previously developed models of glucose-dependent suppression of glucagon secretion have shown that, in hypoglycemia, the amplitude is sufficiently high to cause the opening of P/Q-type Ca^2+^ channels. On the other hand, hyperglycemia leads to faster MP oscillations, but their amplitudes are not sufficient to reach the activation threshold of P/Q channels [5,11,35,51].

Figure 2B,C show the effects of increasing AA levels at low and high glucose concentrations, respectively. While the effects of glucose on MP are opposite and prevent a maximal response of the RGS, this is not true for the effects of AAs on MP. The increase in AA concentration amplifies RGS levels, which the model partly explains by the synchronous increase in both frequency and amplitude of the MP oscillations. The resulting increase in Ca^2+^ entry due to the more frequent and prolonged opening of VGCC leads to a higher intracellular Ca^2+^ concentration, which triggers a maximal RGS response. This effect occurs at both low (Figure 2B) and high (Figure 2C) glucose concentrations, with the effect being more pronounced at the low glucose concentrations.

#### 2.2.2. Effects of cAMP and ATP Concentrations on RGS

**Figure 3:**
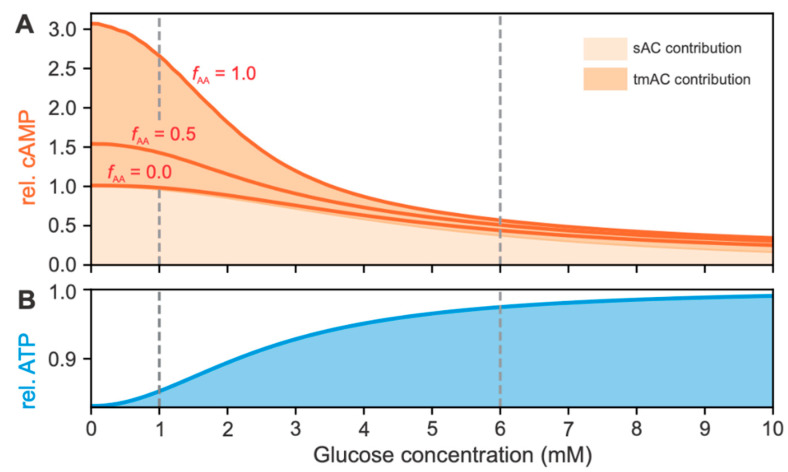
Glucose-dependent intracellular cAMP and ATP concentrations. (**A**) The cAMP concentration is composed of the sAC (light orange) and tmAC (dark orange) contributions. The sAC contribution is invariant with changes in AA concentration. The tmAC contribution increases significantly at low glucose concentration and high AA concentration. (**B**) ATP concentration is only glucose-dependent and increases by ~20% with hyperglycemia.

Increased RGS, due to the intrinsic effects of glucose and AAs on MP dynamics, results in higher interstitial glucagon content, which further stimulates glucagon receptors. These receptors are bound to tmACs, leading to an acceleration of cAMP production. Here, we present the glucose- and AA-induced changes in relative cAMP levels. In addition, the glucose-dependent changes in relative ATP concentration are shown, reflecting the changes in the metabolic component of the model. The glucose- and AA-dependent relative cAMP and ATP concentrations are shown in Figure 3.

Figure 3A shows the glucose-dependent cAMP concentrations at three AA levels ($f_{\mathrm{AA}}$). Coloring under the curves represents the signaling pathway that contributed to the relative cAMP concentration. Light and dark orange colors indicate sAC- and tmAC-induced cAMP concentrations. The results show a ~3-fold increase in tmAC-induced cAMP concentration at the lower end of the physiological glucose concentration, consistent with experimental data on cAMP dynamics in response to glucagon receptor signaling [52,53]. Specifically, concentrations of glucagon corresponding to the EC50 (1.7 nM) of the glucagon-dependent cAMP concentration elicited an approximately 2-fold increase in cAMP response [52]. Since the sAC-induced cAMP production is strongly coupled to alpha cell metabolism, particularly glucose oxidation, AA signaling is independent of the cAMP concentration.

Figure 3B shows the glucose-dependent increase in ATP concentration. The model predicts an increase in ATP concentration of ~20% between 1 mM and 6 mM plasma glucose concentration due to a glucose-dependent increase in glycolytic flux. We have shown that ATP produced from anaerobic glycolysis is necessary for the sufficient increase in ATP level above the glucagon switching point, which occurs at ~6 mM glucose [12].

The cAMP signaling pathway appears to play the dominant role in both glucose- and AA-dependent glucagon signaling. As estimated in our previous work [15], the sAC-dependent increase in cAMP concentration is responsible for most of the intrinsic glucose-dependent change in RGS. More specifically, the results indicated that ~6% change in ATP concentration and ~40% change in cAMP concentration were the energy-driven and signaling-driven switches, contributing ~40% and ~60%, respectively, to RGS. While AAs affect MP oscillations approximately uniformly across all plasma glucose concentrations, high AA levels at low glucose concentrations have the strongest indirect influence on tmAC-derived cAMP concentration. Both effects contribute to the sufficient AA-induced increase in RGS, which is much more pronounced at low glucose concentrations.

## 3. Discussion

As research focuses more on pancreatic alpha cells due to the increasingly recognized importance of glucagon in glucose homeostasis, a growing body of publications provides new insights into the underlying intracellular mechanisms, intra-islet coordination, and role in systemic regulation of metabolites [4,54,55]. While the consensus model for GSGS from alpha cells has yet to be determined, several computational models have been proposed that reproduce experimental results [12,30,31,50,56]. Despite the majority of computational models focusing on glucose-glucagon coupling, it should not be neglected that recent evidence also points to a vital role of alpha cells in systemic regulation of AA metabolism [30,31], with experimental measurements showing a solid response of glucagon secretion to the addition of AAs [29,35].

In this work, we have presented a comprehensive computational model of GSGS that allows simulation of the effects of plasma AA concentration on glucagon secretion and extends our previous modeling of the effects of glucose and fatty acid metabolism on glucagon secretion [12,13,15]. The model predictions successfully reproduce the experimental results of Zhang et al. [35], showing that AAs contribute substantially to the glucagon response of pancreatic alpha cells. An increase in AA levels resulted in a robust maximal glucagon response up to 7-fold stronger than the maximal glucagon response elicited by glucose alone. The mechanisms responsible for this phenomenon are modeled by glutamate-induced modulation of MP oscillations (via iGluR-induced depolarization) and autocrine glucagon-induced stimulation of GcgR-bound tmACs, which increases intracellular cAMP concentration. Model predictions showed that AAs enhance Ca^2+^ entry by synergistically increasing the frequency and amplitude of MP oscillations. However, modulation of MP mainly indirectly affects glucagon exocytosis, whereas most of the net effect is due to increased cAMP concentration.

Systemically, glucagon raises plasma glucose levels [2,57] by acting primarily on the liver glucagon receptors [28,58], counteracting the effects of insulin [59]. However, the systemic role of the strong AA-alpha-cell coupling, also found in the present model, and its role in nutrient homeostasis is not clear but could be related to the need for glucagon secretion during different specific metabolic states [60]. This hypothesis is consistent with clinical data showing that the AA composition of a meal, the form of food intake, the digestibility rate, and the absorption rate have different effects on endocrine responses [61,62]. A critical role of solid AA-alpha-cell coupling may be the adaptation of systemic metabolism to prevent postabsorptive hypoglycemia after a protein-rich meal [63]. Indeed, postprandial metabolism is associated with an increase rather than a decrease in alpha cell activity. The simultaneous AA-induced increase in plasma glucagon levels and insulin levels allows both insulin-induced energy-consuming processes to coincide with the processes of glycogenolysis or gluconeogenesis to prevent hypoglycemia. Considering the crucial role of glutamine in the plasma after food up and digestion [46], the present computational model could have important implications for accurately modeling postprandial glucagon levels.

Apart from preventing postabsorptive hypoglycemia, AA-induced glucagon secretion may also play a role in modulating hepatic gluconeogenesis during fasting, which increases the plasma concentration of various AAs [64]. This transient increase in AAs is likely accompanied by other hormones that counteract insulin, such as cortisol, somatotropin, and thyroid hormones. AA-induced glucagon secretion could signal the liver to further accelerate gluconeogenesis because of the increased availability of AAs, especially alanine, as a gluconeogenic substrate. The prolonged fasting state, in contrast, initiates the AA-sparing mechanisms (such as lipolysis and ketogenesis) to prevent respiratory arrest followed by excessive respiratory muscle atrophy. While the present model may also help distinguish responses to different fasting states, other hormones, such as corticosterone, have been implicated in addition to glucagon in the induction of maximal glucogenesis levels [65].

Although the present study essentially clarifies the principles of AA-induced glucagon secretion and the systemic role of alpha cells in glucose and AA metabolism, the underlying mechanisms are still very elusive. They are controlled by a highly complex and diverse signaling system. To confirm the molecular mechanisms predicted by our computational, experimental studies measuring cAMP and MP dynamics should be conducted. Furthermore, while there is no doubt that AAs play a crucial role in modulating alpha cell activity, several other signals also influence the dynamics of glucagon exocytosis, which we did not consider in the present model. All these aspects should be further investigated in the future, focusing on the cell-to-cell interactions and the integration of humoral and local signals to mimic the exact glucagon response.

## 4. Materials and Methods

**Figure 4:**
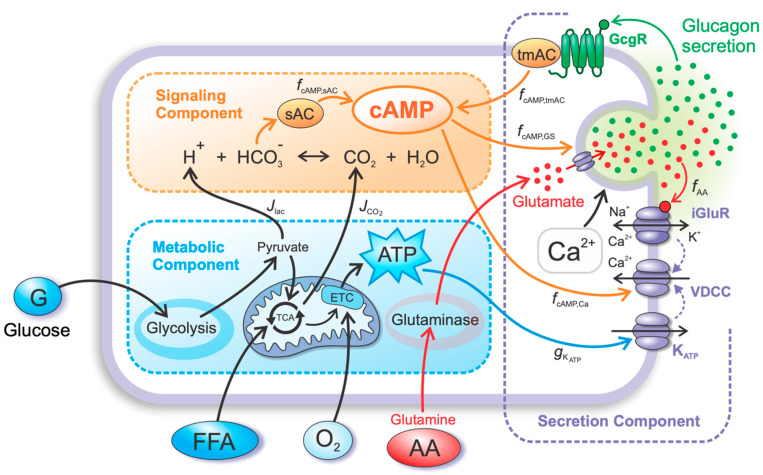
Schematic representation of the computational model. The computational model is governed by three interconnected components—metabolic, signaling, and secretion. The metabolic component is mainly responsible for producing ATP via glucose and FFA oxidation. ATP concentration is coupled to the secretion component via the conductance of K_ATP_ channels. AAs, such as glutamine, which also enter the metabolic component, do not (considerably) contribute to ATP production but can be converted to other types of AAs, such as glutamate, which enters the secretory component. Waste products of metabolism (lactate and CO_2_) interact with the signaling component, influencing the rate of sAC-induced cAMP production. The second contribution to the cAMP concentration is tmAC-induced cAMP production, which is stimulated by the binding of glucagon to the glucagon receptor. The signaling component interacts with the secretory component via cAMP-PKA/Epac signaling pathways, directly and indirectly (via VGCC) influencing glucagon secretion. Finally, glutamate from the metabolic component, which is co-secreted with glucagon, binds to the iGluRs, further modulating glucagon secretion.

The intracellular processes, including the action of AAs and the autocrine action of glucagon on the alpha cell, are implemented based on the previously published models of alpha cell’s metabolic and signaling pathways [12,15], coupled with the model of alpha cell’s electrophysiological properties and glucagon exocytosis by Montefusco et al. [50]. Apart from more precise modeling of alpha cell’s GSGS, these additional mechanisms are certainly important for understanding the nature of the alpha cell’s response to nutrient stimuli other than glucose. The model equations are divided into three distinct but interrelated components (see Figure 4). The first component (called the metabolic component) describes the metabolic response of alpha cells to glucose and FFAs and the resulting increase in CO_2_ and lactic acid, as well as ATP production, leading to an increase in ATP concentration. The second component (signaling component) describes the increase in intracellular cAMP due to the increase in intracellular CO_2_ and lactic acid production. Finally, the third component (secretion component) describes the dynamics of MP and the exocytosis of glucagon granules in response to the conductance of K_ATP_ channels, intracellular cAMP concentration, and extracellular AA concentration.

As illustrated in Figure 4, the three components of the model are described in the continuation: the metabolic, signaling, and secretion components. The corresponding model equations and parameters are presented and related to previous references.

### 4.1. Metabolic Component

The metabolic component describes the catabolic pathways of glucose and FFAs, and the production of ATP by the electron transport chain. These catabolic pathways include glycolysis, beta-oxidation of FFAs, and the tricarboxylic acid (TCA) cycle. In alpha cells, a significant fraction of the glycolysis-derived pyruvate and NADH flux is diverted into lactate production. This anaerobic metabolic pathway appears to be crucial for the physiology of the alpha cell [12,15]. The rate of lactate production ($J_{\mathrm{lac}}$) is described by:

(1) $$J_{\mathrm{lac}}=2p_{L}\;J_{G6P},$$

where $p_{L}$ is the fraction of pyruvate diverted to lactate production and $J_{G6P}$ is the rate of conversion of glucose to glucose-6-phosphate (G6P), a rate-limiting step in glycolysis (for definitions of $p_{L}$ and $J_{G6P}$, see [15]).

The TCA cycle results in the production of CO_2_, the rate of which ($J_{{\mathrm{CO}}_{2}}$) is described by:

(2) $$J_{{\mathrm{CO}}_{2}}=J_{{\mathrm{CO}}_{2},G}+J_{{\mathrm{CO}}_{2},\mathrm{FFA}},$$

where $J_{{\mathrm{CO}}_{2},G}$ is the rate of CO_2_ production by the glucose oxidation pathway and $J_{{\mathrm{CO}}_{2},\mathrm{FFA}}$ is the rate of CO_2_ production by the FFA oxidation pathway. Assuming the respiratory quotient (RQ) for glucose oxidation and FFA oxidation 1 and 0.7, respectively, $J_{{\mathrm{CO}}_{2}}$ can also be expressed by:

(3) $$J_{{\mathrm{CO}}_{2}}=J_{O_{2},G}+0.7\left(J_{O_{2}}-J_{O_{2},G}\right),\;$$

where $J_{O_{2}}$ is the net rate of oxygen consumption and $J_{O_{2},G}$ is the rate of oxygen consumption due to glucose oxidation. The latter expression replaces the influx of FFAs into alpha cell catabolic pathways as the model’s input parameter by the net rate of oxygen consumption. For definitions of fluxes in Equations (2) and (3), see [15].

### 4.2. Signaling Component

The signaling component of the model describes the CO_2_- and lactate-dependent changes in intracellular cAMP concentrations. The mechanism is described in detail in our previous paper [15]. Briefly, intracellular acidification occurs mainly due to the increased rate of lactate production (in response to the increase in extracellular glucose concentration), which perturbs the Henderson-Hasselbalch equilibrium, resulting in lower bicarbonate (${\mathrm{HCO}}_{3}^{-}$) concentration. Consequently, the sACs are less stimulated by the bicarbonate ions, leading to lower cAMP production. At steady-state, cAMP production by the sACs and cAMP hydrolysis by the phosphodiesterases (PDE3B and PDE4, specifically) are in equilibrium, allowing calculation of the absolute cAMP concentration ([cAMP]).

Focusing on the relative effects of [cAMP] on the secretory mechanisms, we introduced the relative cAMP concentration due to sAC stimulation ($f_{\mathrm{cAMP},\mathrm{sAC}}$), as defined in [15]:

(4) $$f_{\mathrm{cAMP},\;\mathrm{sAC}}=\frac{\left[cAMP\right]-{\left[cAMP\right]}_{\min}}{{\left[cAMP\right]}_{\max}-{\left[cAMP\right]}_{\min}}.$$

However, here, we consider not only the stimulation of ubiquitously expressed sACs by bicarbonate ions as the sole source of intracellular cAMP but also the contribution of tmACs, which are located at the plasma membrane and are modulated by the extracellular signals via the G protein-coupled receptors (GPCRs). The tmAC-induced cAMP production and the net intracellular cAMP concentration are described in detail in Section 4.3.2.

As described, cAMP concentration is intrinsically raised due to acidification of the intracellular space and consequent activation of sACs following the increased lactic acid production due to enhanced glucose metabolism. However, intracellular cAMP concentration can also be increased by the binding of extrinsic (autocrine, paracrine, and humoral) signals to the membrane bound GPCRs. Specifically, the glucagon molecule binds to the glucagon receptors, which stimulates the tmACs and leads to the production of cAMP. Considering this, we can divide the effects of relative intracellular cAMP concentration due to intrinsic effects of glucose ($f_{\mathrm{cAMP},\mathrm{intrinsic}})$ and extrinsic effects of extracellular signals ($f_{\mathrm{cAMP},\mathrm{extrinsic}})$:

(5) $$f_{cAMP}=f_{\mathrm{cAMP},\mathrm{intrinsic}}+f_{\mathrm{cAMP},\;\mathrm{extrinsic}},\;$$

where we assume the contribution of each effect to the net relative cAMP concentration to be 75% and 25%:

(6) $$f_{\mathrm{cAMP},\mathrm{intrinsic}}=0.75f_{\mathrm{cAMP},\mathrm{sAC}},\;$$

(7) $$f_{\mathrm{cAMP},\mathrm{extrinsic}}=0.25f_{\mathrm{cAMP},\mathrm{tmAC}}.\;$$

Here, the sAC-derived relative cAMP concentration ($f_{\mathrm{cAMP},\mathrm{sAC}}$) is calculated from the signaling component (Equation (4)), and the calculation of tmAC-derived relative cAMP concentration ($f_{\mathrm{cAMP},\mathrm{tmAC}}$) is presented Section 4.3.2.

### 4.3. Secretion Component

The secretion component incorporates the MP and Ca^2+^ dynamics, which are influenced by the K_ATP_ channel conductance, intracellular cAMP concentration, and extracellular AAs concentration. As in previous models [12,15], we used the model introduced by Montefusco et al. [50] for the secretion component of the model. The time evolution of the MP, considered in the current model, is obtained by solving the voltage equation:

(8) $$\frac{dV}{dt}=-\frac{I_{\mathrm{Na},\mathrm{AA}}+I_{\mathrm{Ca},\mathrm{AA}}+I_{K,\mathrm{AA}}+I_{K_{\mathrm{ATP}}}+I_{L}+I_{\mathrm{SOC}}}{C_{m}},\;$$

where V is the MP and $I_{\mathrm{Na},\mathrm{AA}}$, $I_{\mathrm{Ca},\mathrm{AA}}$, $I_{K,\mathrm{AA}}$, $I_{K_{\mathrm{ATP}}}$, $I_{L}$, and $I_{\mathrm{SOC}}$ are the voltage-gated sodium, voltage-gated calcium, voltage-gated potassium, ATP-dependent potassium, leak, and store-operated currents, respectively, and $C_{m}$ is the membrane capacitance. To account for the effects of cAMP and AAs on the MP, given by Equation (8), we modified the definitions of the ion currents. The changes are described in the next subsection.

#### 4.3.1. The Effects of Glutamate and cAMP on the MP and Glucagon Granule Exocytosis

In the original model given in [50], the voltage-dependent ion currents are independent of the influence of cAMP and other signaling pathways. The alpha cell metabolism and electrophysiology are coupled via K_ATP_ channels conductance ($g_{K_{\mathrm{ATP}}}$), which influences the magnitude of the $I_{K_{\mathrm{ATP}}}$ current and modulates the oscillatory response to the glucose concentration. In our previous model [15], we introduced parameter $f_{\mathrm{cAMP}}$, which modulates the magnitude of the VGCC currents ($I_{\mathrm{Ca}}$).

In the present model, we additionally implemented the concentration-dependent parameter $f_{\mathrm{AA}}$, which occupies values between 0 and 1, representing the relative range of physiological AA levels (as calculated in Results). The stimulatory effect of AAs on glucagon secretion was modeled by considering the binding of the excitatory AA glutamate on the iGluRs [45,66]. It was reported that the glutamate acts on iGluRs of the AMPA/kainate type, resulting in membrane depolarization and consequent increase in cytoplasmic Ca^2+^ concentration [45]. Binding of glutamate to these receptors triggers the opening of cation channels that are permeable to Na^+^, K^+^, and Ca^2+^ ions, which is modeled by the following modifications of cation currents:

(9) $$I_{\mathrm{Ca},\mathrm{AA}}=f_{\mathrm{cAMP},\mathrm{Ca}}\;f_{\mathrm{AA},\mathrm{Ca}}\;\left(I_{\mathrm{CaL}}+I_{\mathrm{CaT}}+I_{\mathrm{CaPQ}}\right),\;$$

(10) $$I_{\mathrm{Na},\mathrm{AA}}=f_{\mathrm{AA},\mathrm{Na}}I_{\mathrm{Na}},\;$$

(11) $$I_{K,\mathrm{AA}}=f_{\mathrm{AA},K}\left(I_{K_{\mathrm{DR}}}+I_{\mathrm{KA}}\right).$$

Here, the indirect effect of cAMP on Ca^2+^ currents ($f_{\mathrm{cAMP},\mathrm{Ca}}$) are modeled by

(12) $$f_{\mathrm{cAMP},\mathrm{Ca}}=\left(1-k_{c\mathrm{AMP},\mathrm{Ca}}\left(1-f_{\mathrm{cAMP}}\right)\right)$$

and the indirect effect of AAs on Na^+^ ($I_{\mathrm{Na},\mathrm{AA}}$), K^+^ ($I_{K,\mathrm{AA}}$) and Ca^2+^ ($I_{\mathrm{Ca},\mathrm{AA}}$) currents via the action of glutamate on iGluR are modeled by:

(13) $$f_{\mathrm{AA},\mathrm{Na}}=1+k_{\mathrm{AA},\mathrm{Na}}\;f_{\mathrm{AA}},$$

(14) $$f_{\mathrm{AA},K}=1+k_{\mathrm{AA},K}\;f_{\mathrm{AA}},$$

(15) $$f_{\mathrm{AA},\mathrm{Ca}}=1+k_{\mathrm{AA},\mathrm{Ca}}\;f_{\mathrm{AA}},$$

respectively. In Equations (13)–(15), parameters $k_{\mathrm{AA},\mathrm{Na}}$, $k_{\mathrm{AA},K}$, and $k_{\mathrm{AA},\mathrm{Ca}}$ reflect the relative effects of iGluR on whole-cell magnitudes of Na^+^, K^+^, and Ca^2+^ currents. The coefficients are defined in Table 1. It was reported that the magnitude of Na^+^ current through iGluR predominates over the magnitude of K^+^ current and that most AMPA receptors have a low permeability for Ca^2+^ cations [67,68,69].

In the present model, we assume $g_{L}$ to be a constant parameter, rather than $g_{K_{\mathrm{ATP}}}$-dependent function (see Table 1):

(16) $$I_{L}=g_{L}\left(V-V_{L}\right)\;$$

The net rate of glucagon secretion, which is a function of $g_{K_{\mathrm{ATP}}}$, $f_{\mathrm{cAMP}}$, and $f_{\mathrm{AA}}$ parameters, is given by:

(17) $$\mathrm{GS}\left(g_{K_{\mathrm{ATP}}};f_{\mathrm{cAMP}};f_{\mathrm{AA}}\right)=f_{\mathrm{cAMP},\mathrm{GS}}\left({\mathrm{GS}}_{L}+{\mathrm{GS}}_{\mathrm{PQ}}\right)+{\mathrm{GS}}_{m},$$

where the direct effect of cAMP on the glucagon secretion ($f_{\mathrm{cAMP},\mathrm{GS}}$) is modeled by:

(18) $$f_{\mathrm{cAMP},\mathrm{GS}}=\left(1-k_{\mathrm{cAMP},\mathrm{GS}}\left(1-f_{\mathrm{cAMP}}\right)\right).\;$$

#### 4.3.2. Autocrine Effects of Glucagon on cAMP Concentration

This subsection addresses the autocrine effects of GSGS and the resulting increase in glucagon concentration in the interstitial space. We assume that the increased interstitial glucagon concentration activates tmACs via glucagon receptor agonism and increases cAMP concentration, which further stimulates glucagon exocytosis. We first define RGS by:

(19) $$\mathrm{RGS}\left(g_{K_{\mathrm{ATP}}};f_{\mathrm{cAMP}};f_{\mathrm{AA}}\right)=\frac{\mathrm{GS}\left(g_{K_{\mathrm{ATP}}};f_{\mathrm{cAMP}};f_{\mathrm{AA}}\right)}{{\mathrm{GS}}_{\mathrm{norm}}},\;$$

where GS is the absolute glucagon secretion rate, defined by Equation (17), and ${\mathrm{GS}}_{\mathrm{norm}}$ is the normalization constant, which is the rate of glucagon secretion in the absence of extrinsic stimuli (glutamate and glucagon) and glucose, defined by:

(20) $${\mathrm{GS}}_{\mathrm{norm}}=\mathrm{GS}\left(g_{K_{\mathrm{ATP}}}^{G=0};\;f_{\mathrm{cAMP},\mathrm{intrinsic}};0\right).\;$$

The glucose- and AA-dependent intrinsic RGS (${\mathrm{RGS}}_{\mathrm{intrinsic}}$), accounting only for the effects of the intrinsically produced cAMP concentration, can then be calculated by:

(21) $${\mathrm{RGS}}_{\mathrm{intrinsic}}=\mathrm{RGS}\left(g_{K_{\mathrm{ATP}}};f_{\mathrm{cAMP},\mathrm{intrinsic}};f_{\mathrm{AA}}\right).$$

${\mathrm{RGS}}_{\mathrm{intrinsic}}$ is used to approximately evaluate the interstitial concentration of glucagon, which is the autocrine signal for the alpha cell. In the final step, the glucagon-induced increase in cAMP concentration via tmACs ($f_{\mathrm{cAMP},\mathrm{tmAC}}$) is calculated from the ${\mathrm{RGS}}_{\mathrm{intrinsic}}$. We model the glucagon concentration-dependent increase in cAMP concentration due to tmAC activation based on the experimental data [52,53] by:

(22) $$f_{\mathrm{cAMP},\mathrm{tmAC}}=\left(1+f_{0}\frac{{\mathrm{RGS}}_{\mathrm{intrinsic}}^{n}}{k^{n}+{\mathrm{RGS}}_{\mathrm{intrinsic}}^{n}}\right){\mathrm{RGS}}_{\mathrm{intrinsic}},$$

where $f_{0}$, K, and n are the fitted parameters, defined in Table 1.

#### 4.3.3. Net Glucose- and AAs-Dependent RGS

Previous sections presented the metabolic component, which yields the intracellular ATP concentration, the signaling component, which considers the calculation of relative cAMP concentration, and the secretion component, introducing the effects of ATP (via $g_{K_{\mathrm{ATP}}}$), cAMP (via $f_{cAMP,Ca}$ and $f_{cAMP,GS}$), and plasma AA concentration (via $f_{\mathrm{AA}}$) on the glucagon secretion. The intrinsic and extrinsic cAMP concentrations are derived by Equations (4) and (22), respectively. The net relative cAMP concentration ($f_{\mathrm{cAMP}}$) is given by Equation (5).

Using the above defined values of $g_{\mathrm{KATP}}$, $f_{\mathrm{cAMP}}$, and $f_{\mathrm{AA}}$, the net RGS (${\mathrm{RGS}}_{\mathrm{net}}$) can be calculated using Equation (19), yielding:

(23) $${\mathrm{RGS}}_{\mathrm{net}}=\mathrm{RGS}\left(g_{K_{\mathrm{ATP}}};f_{\mathrm{cAMP}};f_{\mathrm{AA}}\right),\;$$

Parameter values used in Equations (1)–(23) are listed in Table 1.

## Figures and Tables

**Table 1:** Model parameters and their values.

Parameter	Value
$k_{cAMP,Ca}$	0.2
$k_{cAMP,GS}$	0.5
$k_{AA,Na}$	0.4
$k_{AA,K}$	0.2
$k_{AA,Ca}$	0.16
$g_{L}$	0.25
$f_{0}$	175
k	2.8
n	8

## Data Availability

The source code for the computational model is available as a GitHub repository (https://github.com/janzmazek/Alpha-Beta-Model, accessed on 31 March 2022).
